# Safety and efficacy of the ThermoCool SmartTouch SurroundFlow catheter for atrial fibrillation ablation: A meta‐analysis

**DOI:** 10.1002/clc.23297

**Published:** 2019-11-19

**Authors:** Chao‐Feng Chen, Xiao‐Fei Gao, Mei‐Jun Liu, Chao‐Lun Jin, Yi‐Zhou Xu

**Affiliations:** ^1^ Hangzhou First People's Hospital Zhejiang University School of Medicine Hangzhou City Zhejiang Province China; ^2^ Nanjing Medical University Hangzhou City Zhejiang Province China

**Keywords:** atrial fibrillation, meta‐analysis, radiofrequency ablation, SmartTouch SurroundFlow catheter

## Abstract

**Background:**

The ThermoCool Smarttouch Surroundflow catheter (STSFc) is an advanced catheter, which integrating contact force sensing and surroundflow technology. However, comparative data between STSFc and contact force sensing catheter (Thermocool SmartTouch catheter [STc]) are limited.

**Hypothesis:**

We thought that STSFc might bring more clinical benefits. The aim of this meta‐analysis was to compare the safety and efficiency between the STSFc and the STc for treatment of atrial fibrillation (AF).

**Methods:**

The Medline, PubMed, Embase, and Cochrane Library databases were searched for studies comparing STSFc and STc.

**Results:**

Four trials involving 727 patients were included in the study. Pool‐analyses demonstrated that, as compared STc ablation, STSFc ablation was more beneficial in terms of procedural times (standard mean difference [SMD]: −0.22; 95% confidence interval [CI], −0.37 to −0.07, *P* = .005) and irrigation fluid volume (SMD: −1.94; 95% CI, −2.65 to −1.22, *P* < .0001). There was no significant difference between STSFc and STc (risk ratio [RR]: 1.02; 95% CI: 0.86 to 1.21, *P* = .79) for free from AF. Evidence of complications were low and similar for both groups (RR: 0.83; 95% CI: 0.19‐3.55, *P* = .80). Additionally, patients administered STSFc ablation tended to have shorter fluoroscopic times (SMD: −0.20; 95% CI, −0.63‐0.23, *P* = .21).

**Conclusions:**

STSFc ablation was associated with reducing procedural times and irrigation fluid volume. Further, STSFc ablation tended to shorten fluoroscopic times. Therefore, STSFc ablation would be a better choice for AF patients especially in patients with heart failure.

## 1 INTRODUCTION

Atrial fibrillation (AF) is the most common sustained arrhythmia.^1^ Advances in electro‐physiological technology and increased operator experience over the last decade have permitted catheter ablation to emerge as standard therapy for symptomatic, drug‐refractory AF.^2^, ^3^ However, obtaining long‐term PVs electrical isolation remains challenging with just a single procedure.^4^ A lot of work has been made to develop ablation catheter with the intent of increasing long‐term efficacy and safety of AF ablation.^5^, ^6^, ^7^, ^8^

Open irrigation systems and contact force (CF) sensing represent two important landmarks in ablation catheterization technologies.^5^ In recent years, CF‐sensing catheters have been developed that can directly quantify tissue contact and provide real‐time data to guide ablation. However, earlier irrigation systems of CF catheters utilized a flow system that delivered the irrigating fluid only to the distal surface of the electrode tip, which might result in high volume load and risk of steam pops.^9^ The surround flow (SF) entire tip irrigation system features a wide‐spread distribution of the irrigating solution (56 irrigation holes), which enables homogenous cooling, protection from thrombus formation with lower flow rate requirements, and reduce incidence of steam pops.^6^, ^10^ Both technologies have been widely adopted individually: the Thermocool SmartTouch catheter (STc, Biosense Webster, Diamond Bar) and the ThermoCool SurroundFlow catheter (SFc, Biosense Webster, Diamond Bar, CA). More recently, both technologies were integrated within a single catheter, the ThermoCool SmartTouch SurroundFlow (STSFc, Biosense Webster, Diamond Bar, CA). When compared with STc, STSFc ablation is capable of displaying enhanced clinical benefits, at least in theory. However, clinical data that compares clinical outcomes in the real world between STSFc as compared STc are limited and inconsistent. Thus, we pursue a meta‐analysis to evaluate the clinical benefits of STSFc as compared with STc.

## 2 METHODS

### 2.1 Data sources and search strategy

Relevant articles were searched in the Medline, PubMed, EmBase, Cochrane Library, and Elsevier's ScienceDirect databases. Reports published in non‐English languages were excluded from the search. The search strategy employed relevant keywords and medical subject heading (MeSH) terms including the following: ((atrial fibrillation) OR (AF)) AND ((radiofrequency ablation) OR (RF)) AND ((catheter) OR(SmartTouch SurroundFlow) OR (STSF)) AND ((contact force sensing) OR (Thermocool SmartTouch) OR (ST)). The literature search was updated in August 2019.

### 2.2 Inclusion and exclusion criteria

Two reviewers (C‐CF and G‐XF) screened and identified studies that met the following inclusion criteria: (a) patients with drug‐refractory symptomatic AF that accepted radiofrequency ablation; (b) patients undergoing treatment by catheter ablation for the first time; (c) comparison between STSF catheter ablation and ST catheter ablation; (d) sample size ≥20; and (e) additionally, to be included, studies needed to provide at least one of the reliable information with regard procedure outcomes, complications and follow‐up in both groups. Exclusion criteria were the following: (a) those studies that included the STSFc group with high power RF energy delivery; (b) an equivocal study design or group allocation; and (c) conference abstracts, case reports, case series studies, editorials, review articles, or non‐English language articles.

### 2.3 Quality assessment and data extraction

Study quality was evaluated by two investigators (J‐CL and L‐MJ) using the Newcastle‐Ottawa Quality Assessment Scale (NOS) for observational studies and Delphi consensus criteria for RCTs. The NOS system consists of eight questions with nine possible points. A star system was used to judge the data according to the selected populations, and the comparability of the groups and exposure/outcome of interest. The NOS ≥7 was judged to be a study of good quality.^11^ The preferred reporting items for systematic reviews and meta‐analyses amendment to the quality of reporting of meta‐analyses statement and recommendations from the Cochrane Collaboration in epidemiology were followed during development of the present systematic review. Data extraction was conducted by mutual agreement, and all potential disagreements were solved by consensus.^12^, ^13^

### 2.4 Outcomes definitions

Procedure times: It is the time from the application of local anesthesia to the withdrawal of all catheters.

Ablation times: It is the time from the first to the last application.

Fluoroscopy times: It is the time of fluoroscopy from the start to the end of the procedure.

Irrigation fluid volume: It is the saline irrigation fluid from the catheter at the start to the end of the procedure.

Atrial arrhythmias recrudescence: It is any symptomatic or asymptomatic atrial arrhythmia lasting >30 seconds after completing the blanking period after catheter ablation.

Major complications: It is defined as complications that required any intervention or prolonged hospital stay; other complications were considered as minor complications.

### 2.5 Assessment of heterogeneity reported bias and statistical analysis

A meta‐analysis of the summary statistics from individual trials was performed. Statistical analysis was completed by an independent statistician (C‐CF). Differences in dichotomous variables and outcome endpoints were reported as risk ratio (RR) with 95% confidence intervals (CIs). Continuous variables were analyzed using weighted mean differences or standard mean differences (SMD). Fixed‐and random‐effects models used weighting that was based on an inverse variance, which was calculated according to DerSimonian and Laird.^14^ Between study heterogeneity was reflected by *I* ^2^ > 50%, with a *P* < .05 deemed a statistically significant. When no significant statistical heterogeneity was identified, the fixed effects model was preferentially used as the summary measure. In cases of statistical heterogeneity, sensitivity analyses were performed to assess the contribution of each study to the pooled estimate by sequentially excluding individual trials time and recalculating the pooled RR estimate for the remaining studies.^15^ When pooled analysis still yielded significant heterogeneity, the random‐effects model was used. Statistical analysis was performed using the Review Manager version 5.3 software (The Nordic Cochrane Center, The Cochrane Collaboration, 2014, Copenhagen, Denmark).

## 3 RESULTS

### 3.1 Eligible studies

**Figure 1:**
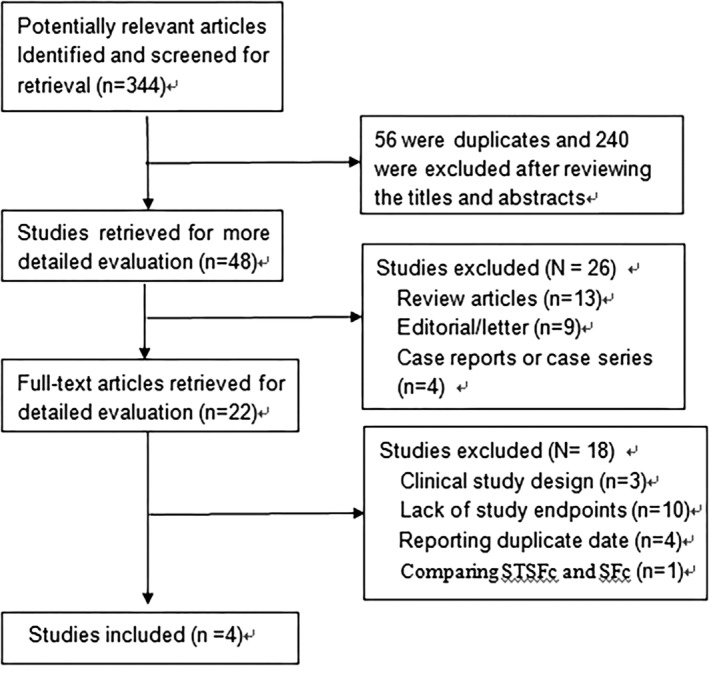
Flow diagram of study selection process

The flowchart of the detailed search process is illustrated in Figure 1. Initially, 344 potentially relevant manuscripts were identified, of which 56 were duplicates and 240 were excluded after reviewing the titles and abstracts. Of the 48 articles that were retrieved for further examination, 13 review articles, 9 editorial/letters, and 4 case reports or case series articles were excluded. Of the remaining 22 studies, 18 were excluded after a detailed evaluation of the full text due to the following: 3, clinical study design; 10, lack of study end points; 4, reporting duplicate date and 1, comparing STSFc and SFc. Finally, 4 clinical trials were included for analysis.

### 3.2 Study characteristics

The characteristics of the four trials are summarized in Table 1. A total of 727 patients were enrolled in these trials (471 were allocated to the STSFc group and 256 were allocated to the STc group). Three of the four trials had a prospective design,^16^, ^17^, ^18^ and most of the trials had patients that were matched on age, gender, body mass index (BMI) and left atrium diameter between the STSFc and STc groups. One study by Chopra et al expounded that patient baseline characteristics and medical history sections were not significantly different between the two groups, but did not show detailed data in the articles.^19^ All studies were rated as having good methodological quality. The results of the grouping ensured the feasibility of this meta‐analysis.

**Table 1:** Baseline characteristics of included study

Author/(Ref.#),year	Country	Treatment Group	Patients	Age (years)	Male (%)	BMI (kg/m^2^)	LA size (mm)	Hy (%)	DM (%)	CAD (%)	Follow	Design	NOS
Plenge T et al (2019)	Germany	STSFc	60	63.0 ± 9.1	63.3	27.4 ± 5.1	41.2 ± 7.0	65	15	20	12 M	Prospectively non‐randomized	9
		STc	20	65.3 ± 10.7	65	25.7 ± 4.3	42.7 ± 6.3	73.3	13.3	40			
Solimene F et al (2019)^a^	Italy multicenter	STSFc	162	60 ± 12	68	27.5 ± 4.3	114 ± 46 ^c^	30.4	11.1	5.3	NR	Prospectively non‐randomized	7
		STc	96	58 ± 10	71	27.2 ± 3.8	95 ± 30^c^	31.3	2.1	3.7			
Solimene F et al (2019)^b^		STSFc	151	59 ± 10	72	26.2 ± 4	79 ± 34^c^	45.7	4	5.5	NR		
		STc	81	59 ± 13	77	28.1 ± 4.8	134 ± 65 ^c^	39.5	2.1	6.2			
Maurer T et al (2018)	Germany	STSFc	75	65.4 ± 11.5	46.7	28.5 ± 6	45.2 ± 6.6	61.3	9.3	29.3	337.2 ± 47.1 D	Prospectively non‐randomized	9
		STc	35	66.6 ± 9	68.6	26.3 ± 4.3	44.23 ± 6	71.4	11.4	22.9	366.8 ± 87.5 D		
Chopra N et al (2018)	USA	STSFc	23	NR	NR	NR	NR	NR	NR	NR	NR	Retrospective	8
		STc	24	NR	NR	NR	NR	NR	NR	NR			

*Note*: Values are reported as the mean ± SD, medians (interquartile range), or n (%).

Abbreviations: BMI, body mass index; CAD, coronary artery disease; D, days; DM, Diabetes mellitus; Hy, hypertension; LA, left atrium; M, month; NOS, Newcastle‐Ottawa Quality Assessment Scale; NR, not recorded; SFc, ThermoCool SurroundFlow catheter; STc, Thermocool SmartTouch catheter; STSFc, ThermoCool SmartTouch SurroundFlow catheter.

a Catheter ablation with AI setting 330 posterior‐450 anterior.

b Catheter ablation with AI setting 380 posterior‐500 anterior.

### 3.3 Procedure outcomes

**Figure 2:**
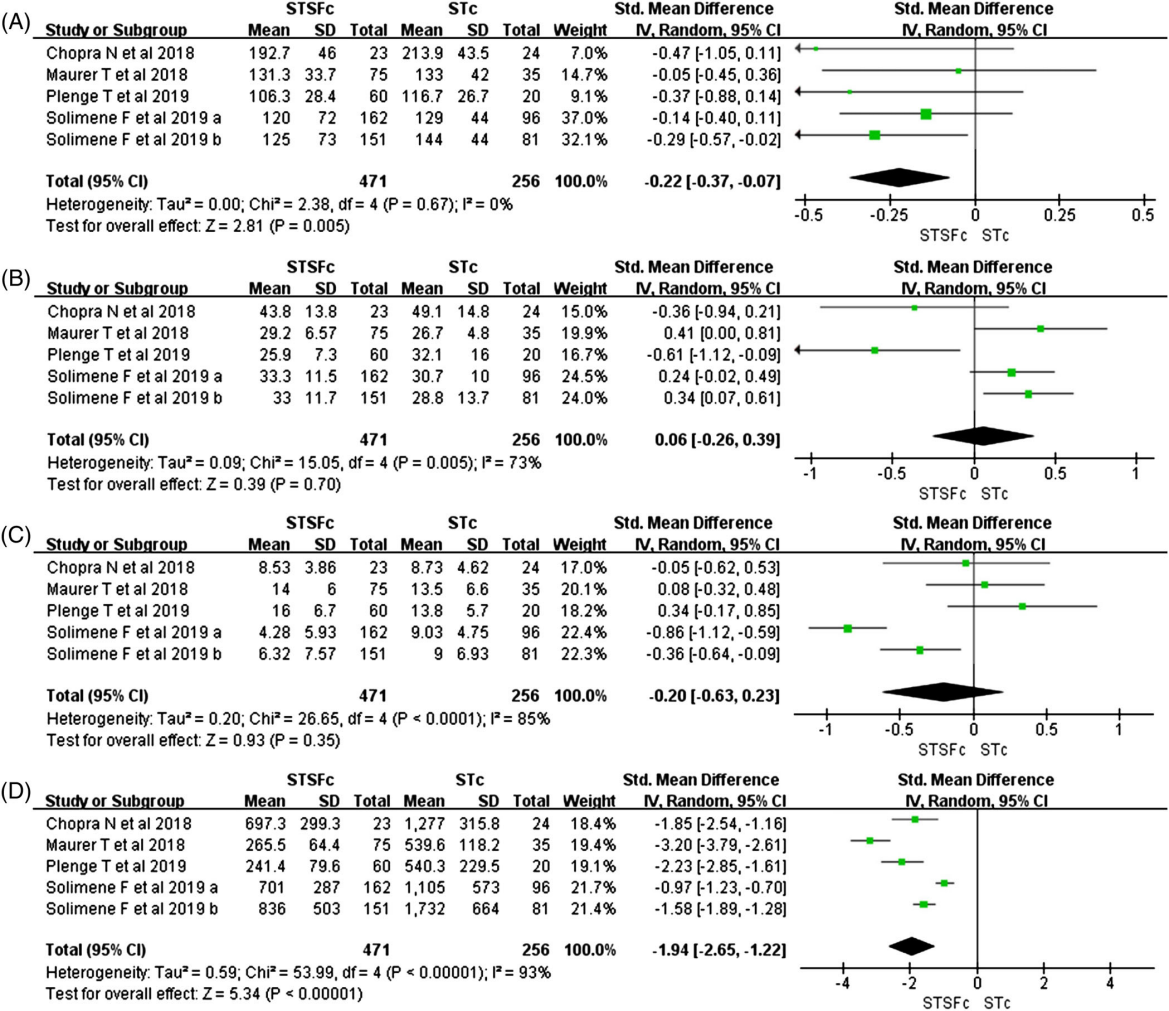
A, Forest plots of procedure times; B, ablation times; C, fluoroscope times and D, irrigation fluid for STSFc vs STc. CI, confidence interval; SD, standard mean difference; STc, Thermocool SmartTouch catheter; STSFc, ThermoCool Smarttouch Surroundflow catheter

The pooled analysis demonstrated that STSFc ablation significantly reduced procedure times (SMD: −0.22; 95% CI, −0.37 to −0.07, *P* = .005; *I* ^2^ = 0%; Figure 2A). No significant differences were observed when comparing STSFc with STc in terms of ablation times and fluoroscopic times (SMD: 0.06; 95% CI, −0.26 to 0.39, *P* = .70; *I* ^2^ = 73%; Figure 2B and SMD: −0.20; 95% CI, −0.63 to 0.23, *P* = .21; *I* ^2^ = 85%; Figure 2C). In open‐irrigated catheters, saline fluid was administered during energy application via the catheter tip into the bloodstream. Compared with STc, saline infusion was significantly lower when the ablation was performed with STSFc (SMD: −1.94; 95% CI, −2.65 to −1.22, *P* < .0001; *I* ^2^ = 93%; Figure 2D).

### 3.4 Complications and free from AF

**Figure 3:**
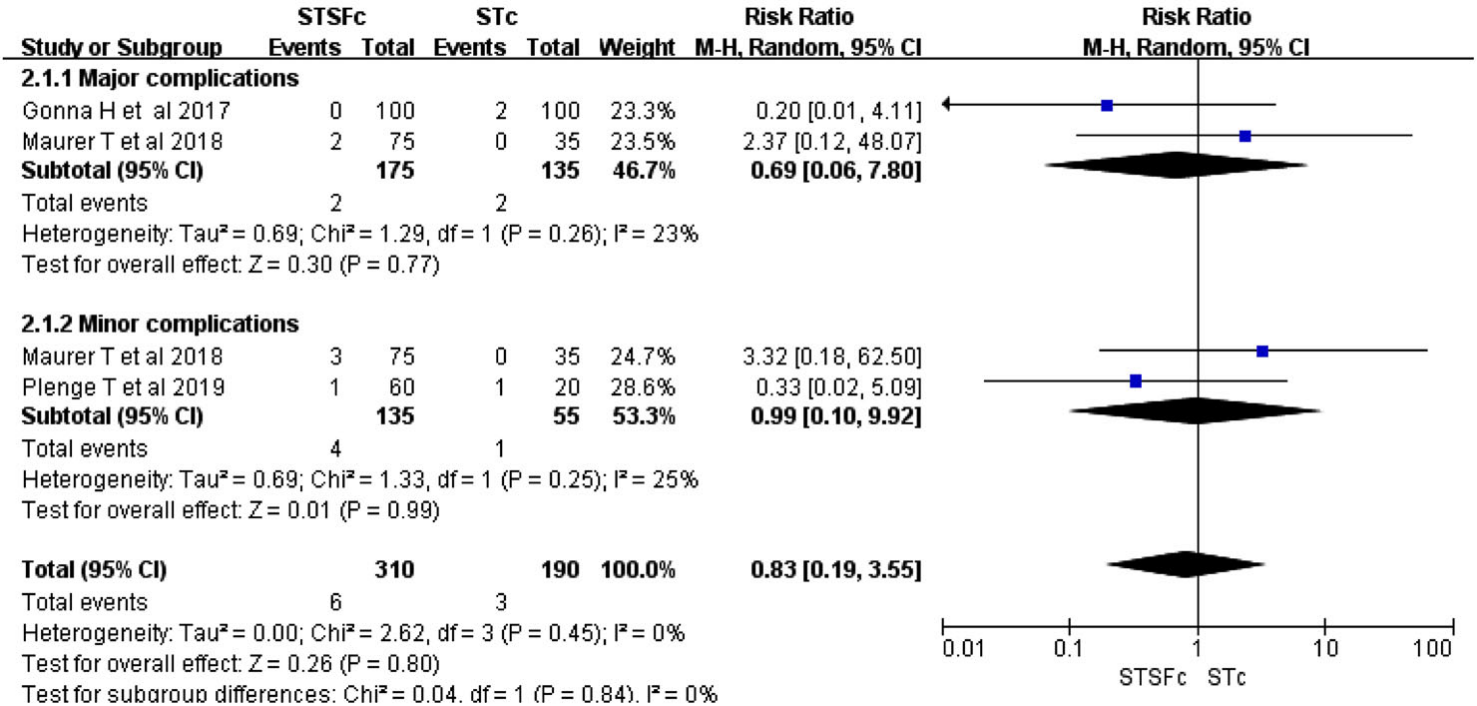
Forest plots of complications for STSFc vs STc. CI, confidence interval; SD, standard mean difference; STc, Thermocool SmartTouch catheter; STSFc, ThermoCool Smarttouch Surroundflow catheter

**Figure 4:**
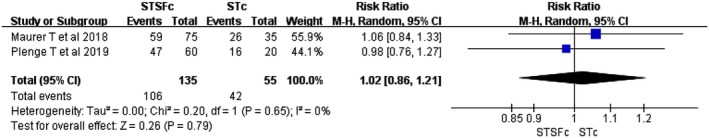
Forest plots of free from AF for STSFc vs STc. CI, confidence interval; SD, standard mean difference; STc, Thermocool SmartTouch catheter; STSFc, ThermoCool Smarttouch Surroundflow catheter

The rate of total complications for both groups was low, and there was no significant differences when comparing STSFc and STc ablation, without heterogeneity (RR: 0.83; 95% CI: 0.19‐3.55, *P* = .80; *I* ^2^ = 0%, *P* = .45; Figure 3). For major complications, there was also no significant difference found when comparing both groups (RR: 0.69; 95% CI: 0.06‐7.8, *P* = .77; Figure 3). Minimal heterogeneity was observed among these studies (*I* ^2^ = 26%, *P* = .26; Figure 3). In addition, the rate of free from AF was similar between STSFc and STc ablation (RR: 1.02; 95% CI: 0.86‐1.21, *P* = .79; *I* ^2^ = 0%; Figure 4).

## 4 DISCUSSION

The present study might represent the first meta‐analysis so far comparing the clinical outcomes between STSFc and STc ablation in patients with AF. The main findings from the pooled analysis were as follows: (a) STSFc ablation can reduce procedure times and irrigation fluid volume as compared to STc ablation; (b) no differences were found in terms of the proportion of patients free from AF between the STSFc and STc groups; (c) STSFc ablation has a tendency to shorten the fluoroscopic times, although the difference is no significant; (d) the ablation times, and complications rates were similar for both groups.

The irrigated ablation catheters (TCc) allows deliver a large amount of RF energy to the tissues, and can prevent the risk of overheating at the electrode‐tissue interface.^20^ The SF entire tip irrigation system improved catheter tip irrigation by increasing the number of irrigation holes from 6 to 56. Several studies found that ablation by SF technology significantly reduces ablation and fluoroscopic times, and early reconnection of PVs when compared with TCc.^6^, ^9^ CF is identified as a key determinant of lesion size, long term ablation efficacy and safety.^8^ A Lot's of observational studies have found overwhelming benefit from CF‐guided AF ablation,^21^, ^22^ however a meta‐analysis of randomized data by Virk et al demonstrated that CF guidance does not improve the safety or efficacy of AF ablation.^23^ Even though, CF sensing technology in STc enables real‐time monitoring of CF between the catheter tip and tissues, and confirmed persistent appropriate pressure to effectively facilitate RF energy transfer to the tissue.^24^, ^25^ Therefore, a combination of SF and CF technologies might further improve procedural success and clinical outcomes. Unfortunately, clinical data on the acute and long‐term success of ablation using SFSFc remains limited. A prospective, open‐label, nonrandomized study (SMART‐SF) was conducted at 17 sites in the US, which might be the first trial on the acute procedural efficacy using STSFc. This trial demonstrated that STSFc was safe and effective for AF ablation, with an acute PVI rate of up to 96.2%. However, the data suffers from lacking any long‐term follow‐up outcomes.^7^ Stabile et al reported a multicenter trial on the safety and efficacy of STSFc for patients with AF, wherein it was found that STSFc achieved a high rate of PVI (99%), and the procedural and fluoroscopic times were comparable with other catheters and with a low complication rate. Moreover, the midterm success rates reached almost 90%.^26^ Thus, STSFc represents an ideal ablation catheter for AF ablation. However, whether STSFc can promote additional clinical benefit when compared with STc requires formal demonstration and is unknown.

Recently, some comparisons between STSFc and STc were reported. In the SMART‐SF trial, procedure features and fluid delivery by STSFc were observed in comparison to predecessor catheters from other registration trials. They found that average procedural times, fluoroscopic times and saline infusion volumes were significantly lower than reported in previous registration trials.^7^ In addition, Gonna et al, showed a reduced rate of primary composite endpoints of procedure failure and acute complications for STSFc in a matched pair analysis of STSFc as compared SFc.^5^ Moreover, Plenge et al presented the first prospective trial and controlled data comparing STSFc with STc. It was found that when using STSFc, this resulted in shorter ablation times; additionally, the rate of recrudescence and complications were similar for both groups.^16^

In an additional prospective trial by Maurer et al, it was shown that 79.7% of patients from the STSFc group were free from AF (74.3% in the STc group) after a mean follow‐up of 12 ± 3 months, even though the result did not reach statistical significance (*P* = .18), there was a trend towards improved efficacy of the STSFc when compared to STc.^18^ Additionally, the Ablation Index (AI), which represents a new lesion quality marker, has been shown to allow acute durable PVI followed by increased long‐term success.^27^, ^28^ Solimene et al made a prospective, multicenter study and compared STSFc with STc for AF ablation that was guided by different AI settings. They showed that STSFc significantly reduced both procedure and fluoroscopic times even with different AI settings.^17^

In the present study, the pooled analysis showed that STSFc was associated with a significant decreasing procedure times, which was consistent with previously published studies. For fluoroscopic times, there was a trend for reductions in the STSFc group, but the difference was not significant. The rate of total complications was low and similar in both groups. For the subgroups, we also found that major or minor complications were similar. No patients experienced atrial‐esophageal fistulas or deaths. Additionally, studies that presented long‐time follow‐up that compared STSFc and STc were limited. Only two studies were included in our analysis, and the results showed no differences in terms of long‐term success rate between the STSFc and STc groups. One reason for this included CF‐sensing technology would be the most important factor contributing to the superior success rate, which enables real‐time monitoring, and ensures stable contact, and more RF energy delivery to the tissues that collectively create a deep and durable tissue lesion.^29^ The other possible reason: SF technology might not improve the long‐term success significantly. This hypothesis was supported by some clinical trials. Bertaglia et al reported a RCT study between SFc and TCc, and observed a similar 6 months follow‐up success rate between for both groups, which finding was in line with Park et al.^6^, ^9^ The possible mechanism might have involved an observation of no significant differences in regard to RF power delivery or lesion size between SFc and TCc.^30^ Therefore, both STSFc and STc share CF‐sensing technology, which results in similarly long‐term success rates between two groups.

Some studies demonstrated that catheter ablation of AF in patients with heart failure (HF) is associated with a significantly lower rate of composite endpoints of death from any cause or hospitalization for worsening heart failure as compared to medical therapy.^31^, ^32^ However, cooled tip catheter use an open irrigation technology resulting in delivery of irrigation fluid into the blood stream. In TCc or STc, an irrigation flow rate of 17 mL/h is recommended if RF power is limited to 30 W. Exceeding 30 W, an irrigation rate of 30 mL/h is necessary. Thus, this population is at risk of acute worsening of HF due to excessive fluid delivery during the procedure. The STSFc procedure uses more homogeneous cooling of the whole tip, so less irrigation fluid is needed. In our study, we found that STSFc had a statistically significant reduction in administered irrigation fluid, which was in line with previous studies. Therefore, STSFc might represent an especially safe and useful approach when treating patients with impaired ventricular function.

Moreover, some studies have shown that high‐power and short‐ablation duration (HPSD) is safe and results in excellent long‐term freedom from AF with short procedure times.^33^, ^34^ In order to reduce the data bias, studies of HPSD using STSFc as compared with other catheters were excluded from our analysis; however, it is undeniable that, STSFc is a suitable catheter for HPSD. Dhillon et al has reported a prospective, multicenter trials that compared STSFc with HPSD and STc with conventional powers. They found that HPSD using STSFc is safe and led to shorter procedure times with a reduced acute PV reconnection as compared to conventional ablation.^35^ Thus, the safe and efficacious application of HPSD using STSFc as identified by more clinical trials, informs us that STSFc might be widely applied in the future for AF ablation.

### 4.1 Study limitations

This meta‐analysis has several limitations: First, publication bias could not be completely excluded, as with any literature search of databases, and inclusion of only published data contributed to bias; Second, the numbers of included studies was limited to only five trials; Third, most of the studies were designed as prospective, non‐randomized trials. Thus, more well‐designed and large‐scale RCTs are required to confirm the findings; Fourth, in the context of important clinical outcomes from complications and long‐term follow‐up, the included studies, we acknowledge that fewer studies had reported the related end‐points, which made our pooled analysis relatively weak.

## 5 CONCLUSIONS

The present systematic review and meta‐analysis demonstrated that STSFc appears to be related to a shorter procedure times than STc, and has a tendency to shorten fluoroscopy times. Compared with STc, STSFc significantly reduces irrigation fluid. Thus, for AF patients, and especially those with HF, STSFc would be a better choice. In addition, STSFc is as effective as STc in terms of long‐term success rates for treating AF. Moreover, STSFc may be best applied under situations where the safety and efficacy of HPSD is confirmed by in future studies.

## CONFLICT OF INTEREST

The authors declare no potential conflict of interests.

## AUTHOR CONTRIBUTIONS

Dr Chao‐feng Chen and Dr Xiao‐Fei Gao: contributed to design of this work, statistical analysis and wrote the article, Mei‐jun Liu: contributed to evaluate quality and retrieved the required data, Chao‐lun Jin: contributed to reviewed the literature, performed the selection of the studies and helped gather references for the article. Dr Yi‐zhou Xu: contributed to design of this work, revised and approved the final version of the article.

## ETHICS STATEMENT

Ethical approval was not necessary to conduct this study.
